# How rapidly do self‐compatible populations evolve selfing? Mating system estimation within recently evolved self‐compatible populations of Azorean *Tolpis succulenta* (Asteraceae)

**DOI:** 10.1002/ece3.6992

**Published:** 2020-11-20

**Authors:** Benjamin Kerbs, Daniel J. Crawford, Griffin White, Mónica Moura, Lurdes Borges Silva, Hanno Schaefer, Keely Brown, Mark E. Mort, John K. Kelly

**Affiliations:** ^1^ Department of Ecology & Evolutionary Biology University of Kansas Lawrence KS USA; ^2^ Biodiversity Institute University of Kansas Lawrence KS USA; ^3^ ETH Zurich Functional Genomics Center Zurich Zurich Switzerland; ^4^ InBIO Laboratório Associado, Pólo dos Açores Faculdade de Ciências Tecnoclogia CIBIO, Centro de Investigação em Biodiversidade e Recursos Genéticos Universidade dos Açores Ponta Delgada Portugal; ^5^ Department of Ecology and Ecosystem Management Plant Biodiversity Research Technical University of Munich Freising Germany

**Keywords:** island colonization, mating systems, self‐incompatibility, *Tolpis succulenta*

## Abstract

Genome‐wide genotyping and Bayesian inference method (BORICE) were employed to estimate outcrossing rates and paternity in two small plant populations of *Tolpis succulenta* (Asteraceae) on Graciosa island in the Azores. These two known extant populations of *T. succulenta* on Graciosa have recently evolved self‐compatibility. Despite the expectation that selfing would occur at an appreciable rate (self‐incompatible populations of the same species show low but nonzero selfing), high outcrossing was found in progeny arrays from maternal plants in both populations. This is inconsistent with an immediate transition to high selfing following the breakdown of a genetic incompatibility system. This finding is surprising given the small population sizes and the recent colonization of an island from self‐incompatible colonists of *T. succulenta* from another island in the Azores, and a potential paucity of pollinators, all factors selecting for selfing through reproductive assurance. The self‐compatible lineage(s) likely have high inbreeding depression (ID) that effectively halts the evolution of increased selfing, but this remains to be determined. Like their progeny, all maternal plants in both populations are fully outbred, which is consistent with but not proof of high ID. High multiple paternity was found in both populations, which may be due in part to the abundant pollinators observed during the flowering season.

## Graphical abstract

Genome‐wide genotyping and Bayesian inference method (BORICE) were employed to estimate outcrossing rates and paternity in two small plant populations of Azorean Tolpis that have recently evolved self‐compatibility. Surprisingly, the populations are highly outcrossing, suggesting that neither a paucity of compatible mates or pollinators are selecting for selfing and that strong inbreeding depression may select against selfing.

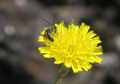

## 1 INTRODUCTION

The evolution of self‐fertilization (selfing) is one of the most common trends in flowering plants (Barrett, 2013; Grossenbacher et al., 2017; Stebbins, 1957; Wright et al., 2013). The first step in this transition is a change in the breeding system with the loss of genetic self‐incompatibility (SI). This loss, often considered a unidirectional transition (Barrett, 2013; Goodwillie, 1999; Herman & Schoen, 2016; Layman et al., 2017; Wright et al., 2013), has pronounced consequences for the subsequent evolution of a species. Most obviously, the loss of SI enables a change from a highly outcrossing mating system (who mates with whom and how frequently in populations) to the potential for higher rates of self‐fertilization, with cascading effects on the distribution of genetic variation among individuals and populations, and on the balance between mutation and natural selection (Kelly, 1999; Lande & Schemske, 1985). Microevolutionary changes have macroevolutionary consequences (Cheptou, 2018; Igić & Busch, 2013). Comparative studies in at least four plant families have shown that self‐compatible (SC) lineages have higher extinction rates and lower net‐diversification rates than closely related SI lineages (Freyman & Höhna, 2018; Gamisch et al., 2015; Goldberg et al., 2010; de Vos et al., 2014).

**FIGURE 1:**
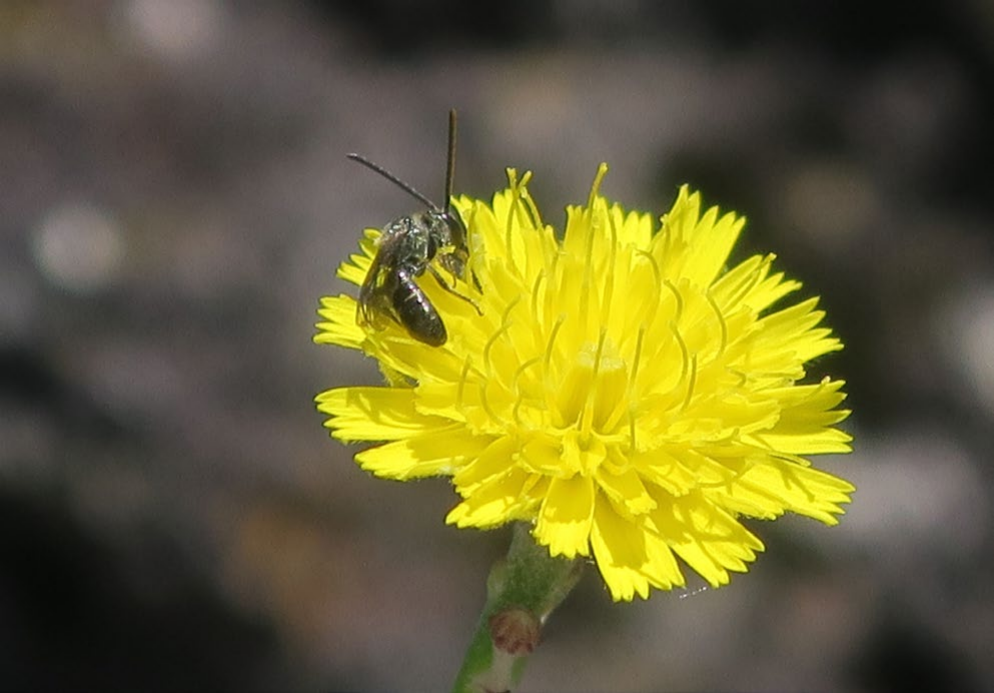
Capitulum of*Tolpis succulenta*on Graciosa Island, visited by a small halictid bee (*Halictus malachurus*). Photo by H. Shaefer

Comparative studies do not indicate how rapidly the loss of SI leads to mating system change. Natural populations routinely harbor genetic variation in reproductive traits that determine selfing rate and thus have the capacity to rapidly evolve selfing (e.g., Bodbyl‐Roels & Kelly, 2011; Thomann et al., 2013). However, ecological and selective factors may prevent a rapid transition. Selfing is favored both by reproductive assurance and automatic selection. The latter refers to the 3:2 transmission advantage enjoyed by selfers when they fertilize their own ovules as well as outcrossing (Fisher, 1941; Jain, 1976; Pannell & Voillemot, 2017). While these are strong forces, high outcrossing can be maintained if selfed progeny are much less fit than outcrossed progeny (inbreeding depression, hereafter indicated as ID, Arista et al., 2017; Husband & Schemske, 1996), if mates are abundant, or if factors like pollen or seed discounting undermine automatic selection. In this study, we examine the tempo of selfing transitions by estimating the mating system in recently evolved SC populations of *Tolpis succulenta* (Asteraceae: Cichorieae). This is a species with ecological and demographic characteristics that should rapidly select for high selfing (Figure 1).

The genus *Tolpis* (Asteraceae: Cichorieae) is a small monophyletic group occurring mainly in the Macaronesian archipelagos, especially the Canary Islands (Jarvis, 1980; Mort et al., 2015). The breeding system of *Tolpis* is overwhelmingly SI with little or no self‐seed produced in most populations (Crawford et al., 2008, 2015). However, SI systems can be “leaky” (pseudo‐self‐compatible or PSC; Levin, 1996) and greenhouse experiments on *Tolpis* plants from some populations can produce low levels of self‐seed when denied cross‐pollen (Crawford et al., 2008, 2015). In this paper, we focus on *T. succulenta* sensu lato, a species that was described from Madeira, but later considered to also occur in the Azores (Jarvis, 1980). The Azorean plants are morphologically distinct from the Madeiran plants, and the two archipelagos are highly divergent at SSR molecular markers (Borges Silva et al., 2015). Lastly, they form distinct clades with genomic markers (Crawford et al., 2019; Kerbs, 2020), indicating that the Azorean plants likely represent a distinct species. Azorean *T. succulenta* are known from very few populations in rocky areas and coastal cliffs on several islands (Jarvis, 1980; Schaefer, 2005). The breeding system of Azorean *T. succulenta* is considered largely SI or PSC based on lack of or very low self‐seed set from two populations on two islands in the Azores (Crawford et al., 2015). Recent mating system estimation using field collected progeny sets from two populations on Madeira confirm predominant outcrossing (Gibson et al., 2020). However, despite a functional SI system, two of the 75 offspring scored in that study were confirmed as products of self‐fertilization (Gibson et al., 2020).

**FIGURE 2:**
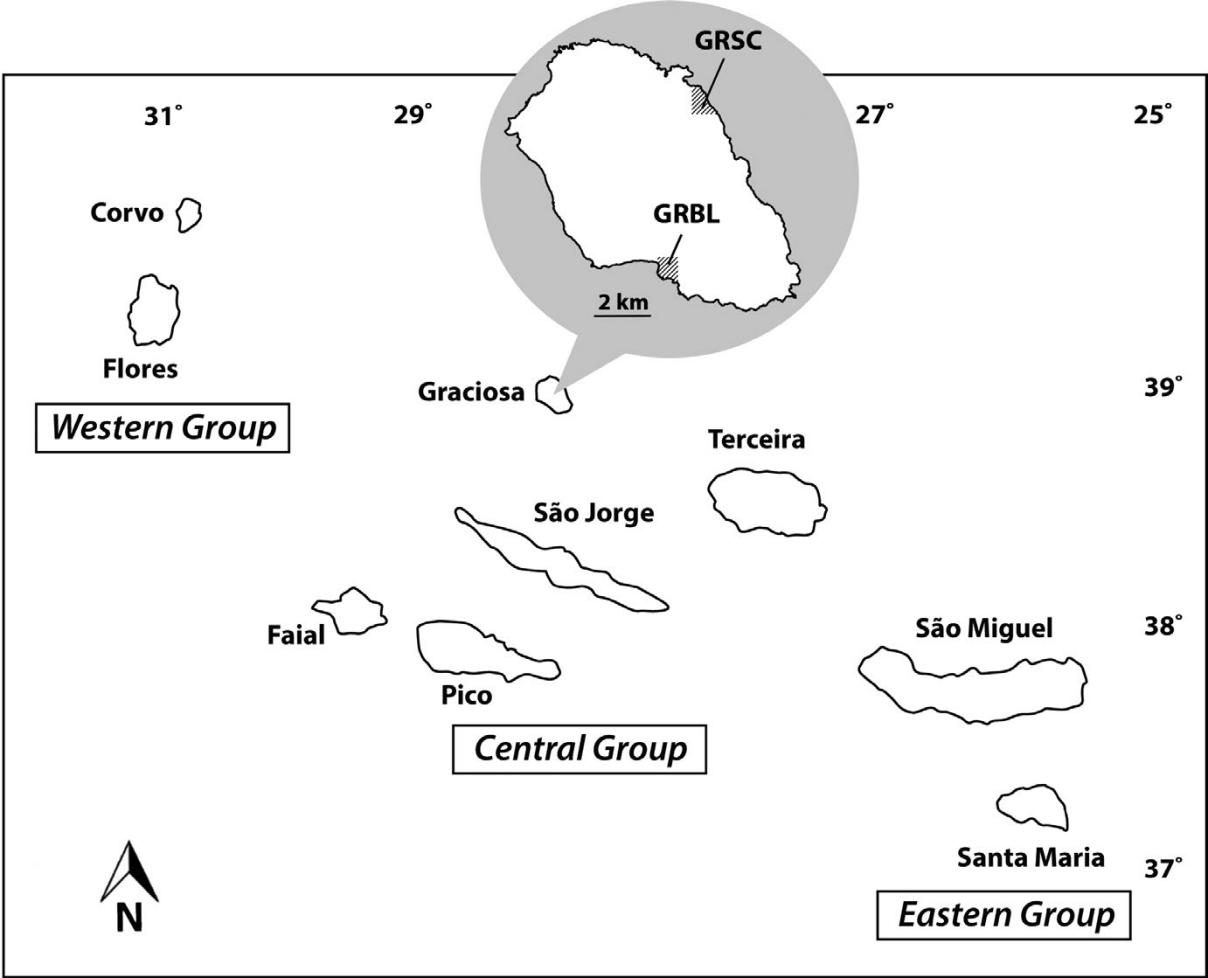
Locality of two populations of*Tolpis succulenta*(GRSC and GRBL) on Graciosa island, within the central group of the Azorean archipelago

Crawford et al. (2019) recently documented a breeding system shift from SI to SC within *T. succulenta* from the Azorean island of Graciosa (Figure 2). In contrast to Madeiran *T. succulenta* and populations from other islands in the Azores, plants from Graciosa readily self in the greenhouse. This shift has a genetic basis: self‐seed set segregates in a nearly Mendelian fashion in F_2_ hybrids between Graciosa plants and SI *T. succulenta* (J. K. Kelly et al., unpubl). As a contrast to *T. succulenta* in Madeira, we here estimate the outcrossing rate in the field for the two known populations on Graciosa (GRSC and GRBL; Figure 2). These populations are very small, with estimated census sizes of 30–80 (GRBL) and 10–20 individuals (GRSC), respectively. Judging from the few collections of *T. succulenta* made from Graciosa, it appears that flowering can occur from July through September, which falls within the broad flowering period (June to September) for SI populations of Azorean *T. succulenta* (Jarvis, 1980; Schaefer, 2005). Both populations occur in disturbed habitats (Crawford et al., 2019; L. Borges Silva & M. Moura, unpubl.). GRSC and GRBL are the only populations of *T. succulenta* in the Azores where SC has been documented (although one or several very small SC populations may occur on Corvo Island; H. Schaefer, pers. obs.). These two populations are sister and form a strongly supported clade in a molecular phylogeny of Azorean *T. succulenta* (Crawford et al., 2019). Field studies conducted on Graciosa after this manuscript was accepted indicate that population GRSC may be extinct, as plants could not be located (H. Schaefer, pers. obs. 2020). The floral parts in plants from GRSC and GRBL are smaller than in SI *T. succulenta* (Crawford et al., 2019; L. Borges Silva & M. Moura, unpubl.) but the “selfing syndrome” (Cutter, 2019; Ornduff, 1969; Slotte et al., 2012) is not nearly as highly developed as in ostensibly more ancient transitions to predominant selfing in Macaronesian *Tolpis* (Crawford et al., 2008; Koseva et al., 2017; Soto‐Trejo et al., 2013).

Another line of evidence supports Graciosa *T. succulenta* as a recent origin of SC. The transition to selfing was likely associated with the colonization of disturbed habitats (complex volcanic history and/or human colonization) on Graciosa. If so, the loss of SI occurred in the range of 400,000 and 1.05 million years as estimated by several radiometric dating studies (Larrea et al., 2014) or 700,000 years as dated by Sibrant et al. (2014) for the age of Graciosa. Secondly, the estimated divergence time between SI and SC *T. succulenta* in the Azores based on a dated Bayesian tree using genome‐wide genotyping is 511,000 years (B. Kerbs et al., unpubl.). The range of island age estimates from radiometric dating, estimated divergence time from a dated molecular phylogeny, and the limited evolution of the floral selfing syndrome point to a recent origin of SC in Graciosa succulenta (Crawford et al., 2019). We hypothesized that the transition to SC could lead to high selfing in GRSC and GRBL because of the limited number of compatible mates and pollinators in small populations, which should produce strong selection for reproductive assurance (Pannell, 2015). Alternatively, ID could select against selfing and maintain outcrossing.

## 2 MATERIALS AND METHODS

### 2.1 Sampling

This study examined the two known populations of *T. succulenta* (herein labeled GRBL and GRSC) which occur on Graciosa island in the Azores (Figure 2): (a). GRBL, Baía do Filipe, Beira Mar da Luz, N 39 01′14.6″, W 28 00′26″, 10 m a. s. l. *Moura GRBL*; (b) GRSC Santa Cruz, Quitadouro, N 39 04′37.8″, W 27 59′19.1″ ca 65 m a. s. l. *Moura*. GRBL, occurring on the southern side of Graciosa in costal basaltic rocks and sheltered by a rock wall, is comprised of ca. 30 (H. Schaefer, pers. obs. 2018) to 80 individuals (Parque Natural de Graciosa, staff pers. obs. 2018). GRSC, occurring on the northern side of the island on an ignimbrite wall on the slope of a volcano, has a population size between 10 (H. Schaefer, pers. obs.) and 20 plants (Parque Natural de Graciosa staff, personal observation). These two populations are separated by about 6 km, and appear to be the only extant ones on Graciosa island. Two other small populations are now presumed to be extinct, having not been located in recent field studies (Parque Natural da Graciosa staff personal observation in 2018 and H. Schaefer in 2018; discussed in Crawford et al., 2019). A “synthetic” population was established from plants of unknown origin on Graciosa that was established between 2001 and 2003 on the small islet of Ilhéu da Praia located, which is located 1.5 km off the eastern coast Graciosa. It is possible that a population occurs on two very small islets (Ilhéu de Baixo) some 700 m off the SE coast of Graciosa (see discussion and map in Crawford et al., 2019). Fruits from capitula of four maternal plants in each population were collected in 2015 and sent to the University of Kansas for cultivation. A total of 22 progeny (2–7 progeny per mother plant from population GRBL and 20 progeny (2–7 per mother plant) from GRSC were genotyped (Table 1). Mean and range of greenhouse self‐seed set in the two populations were 60% (31%–96%) for GRBL and 41.9% (0%–99%) for GRSC (Crawford et al., 2019). Vouchers of progeny are deposited in the R. L. McGregor Herbarium of the University of Kansas (KANU).

**TABLE 1:** Assignment of sibship for each family across two *Tolpis* populations

Population	Family	No. of offspring	No. of sires	Sibship
GRBL	Family 1	7	6	Offspring 3 & 7 full sibs
GRBL	Family 2	2	2	No full sibs
GRBL	Family 3	6	6	No full sibs
GRBL	Family 4	7	4	Offspring 3, 4, 6, & 7 full sibs
GRSC	Family 5	7	3	Offspring 1, 6, & 7 full sibs; 4 & 5 full sibs
GRSC	Family 6	6	6	No full sibs
GRSC	Family 7	2	2	No full sibs
GRSC	Family 8	5	5	No full sibs

### 2.2 Cultivation and DNA extraction

Seeds from wild maternal plants were germinated and reared in greenhouses at the University of Kansas. Leaf tissue was collected, pressed, dried, and ground. Samples were then frozen using liquid nitrogen and pulverized using chromium beads. DNA was subsequently extracted from the ground, dried tissue using DNeasy Plant Mini Kits (Qiagen Inc.) and DNA quantity was validated using a Qubit fluorometer (Thermo Fisher Scientific).

### 2.3 DNA sequencing and SNP calling

RADseq libraries were constructed following the multiplexed shotgun genotyping (MSG) protocol (Andolfatto et al., 2011). DNA from samples was cut using the restriction enzyme Csp6I (syn. CviQI), 250–300 bp fragments were selected for using a BluePippin (Sage Science), and 6 bp barcodes were ligated to fragments. DNA was sequenced on an Illumina NovaSeq 6000 (Novogene) to produce 150 bp paired‐end reads. Following sequencing, the demultiplexing of FastQ files was carried out using STACKS (Catchen et al., 2013) and loci were de novo assembled using the same program with parameters *M* = 2, *m* = 3, *n* = 1, as well as invoking the deleveraging algorithm and specifying alpha = 0.05 in ustacks. De novo assembly and SNP calling yielded a total of 111,613 variant sites.

### 2.4 Mating system estimation and multiple paternity

The resultant VCF from STACKS was assessed, and SNPs were filtered using custom python scripts. We first determined the distribution of reads per SNP per individual at all SNPs called in at least 10 plants. SNPs called in fewer than 10 plants were suppressed. We next eliminated SNPs with excessively high or low coverage: low threshold = 7.4 based on 10th percentile of distribution, high threshold = 42 based on 90th percentile. 8,530 SNPs remained. We then suppressed SNPs that exhibited a statistically significant excess of heterozygotes (relative to Hardy–Weinberg proportions) and then thinned to the data to one SNP per RADtag. We selected the one SNP per RADtag with the most minor genotype calls. This produced the list of 516 SNPs that were formatted for input to BORICE. We simultaneously made the “CX” file that species the fraction of SNPs called for each plant, an input to the genotype uncertainty calculations in BORICE. We first determined the distribution of reads per SNP per individual at all SNPs called in at least 10 plants. SNPs called in fewer than 10 plants were suppressed. We next eliminated SNPs with excessively high or low coverage: low threshold = 7.4 based on 10th percentile of distribution, high threshold = 42 based on 90th percentile. 8,530 SNPs remained. We then suppressed SNPs that exhibited a statistically significant excess of heterozygotes (relative to Hardy–Weinberg proportions) and then thinned to the data to one SNP per RADtag. We selected the one SNP per RADtag with the most minor genotype calls. This produced the list of 516 SNPs that were formatted for input to BORICE. We simultaneously made the “CX” file that species the fraction of SNPs called for each plant, an input to the genotype uncertainty calculations in BORICE. The programs used to perform these operations as well as the genotype file and BORICE settings script are contained in Supplemental File 1.

The BORICE program (Colicchio et al., 2020) uses MCMC (Metropolis et al., 1953) to (a) estimate population outcrossing rates, (b) estimate maternal plant inbreeding coefficients, (c) ascertain whether progeny are selfed or outcrossed, and (d) assess single or multiple paternity of offspring (Gibson et al., 2020). BORICE was run with burn‐in of 1,000 and chain length of 4,000 steps.

## 3 RESULTS

### 3.1 Inbredness of individual parents and offspring

**FIGURE 3:**
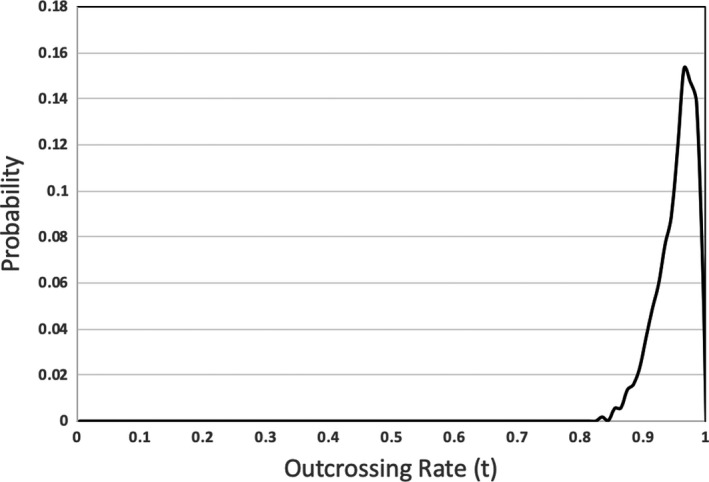
Posterior density for the overall outcrossing rate (*t*) across both populations

Individual progeny from both populations were determined to be outcrossed or selfed with strong confidence (posterior probabilities > 0.99). One offspring of the five in family 8 from GRSC was found to be selfed; hence, the posterior probability for the overall outcrossing is 0 at *t* = 1 (Figure 3). All other offspring were found to be produced via outcrossing. All eight maternal plants across the two populations were determined to be outbred. The high posterior probabilities—P[IH = 0] = 100% for families 1, 3, 4, 5, and 6, 95% for family 2, 94% for family 7, and 81% for family 8. There was minimal (0.05) allele frequency divergence between populations at the markers used for BORICE.

### 3.2 Sibships

**FIGURE 4:**
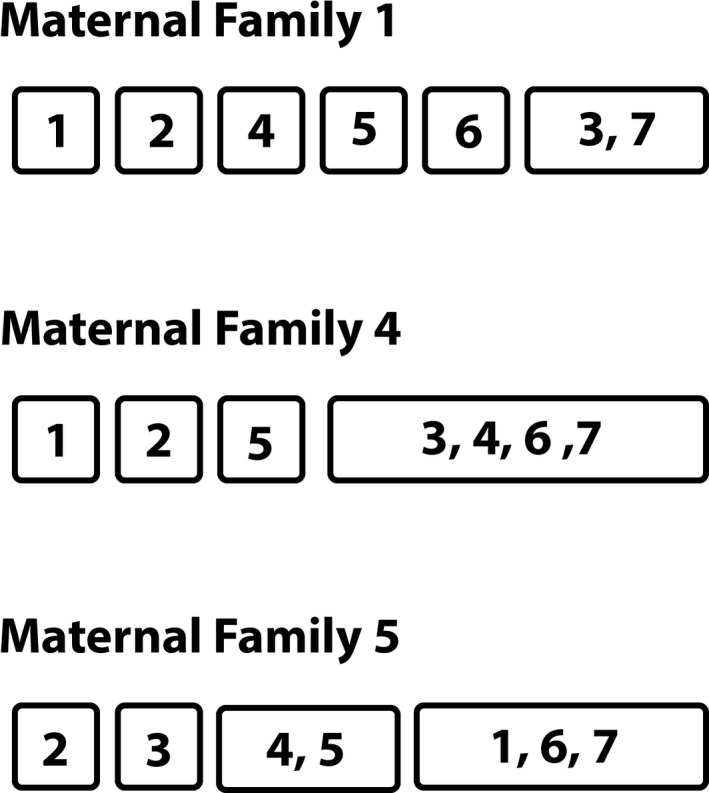
Individual offspring within the same rectangles are full sibs. Maternal family 1 has six sires, and families four and five have four sires. As shown in Table 1, the other five families have no full sibs

Across all families, the probability that progeny are full sibs is 15.3%. No full siblings were detected in five of the eight families (Table 1). Families 1 and 4 contain one set of full sibs each, and family 5 contains two sets of full sibs (Table 1; Figure 4). Outputs show a very high confidence (>90%) in assignment of offspring to a sire in the vast majority (78%) of contrasts. There was moderate support (~50% to 90% probability) for 23% of contrasts.

## 4 DISCUSSION

The loss of genetic SI allows mating system change, but it does not compel the evolution of predominant selfing as an immediate outcome. Numerous genera and families of Angiosperms contain mostly outcrossing species despite the absence of a genetic SI system. Indeed, many of these clades (e.g., *Mimulus*: Phrymaceae) contain recently evolved selfing species, which indicates that selfing can evolve, but the bulk of species remain outcrossing. The continued persistence of the outcrossing species indicates that microevolutionary processes can maintain outcrossing with SC lineages over (relatively) long time scales. Here, we find that *Tolpis* lineages that have recently evolved SC were highly outcrossing, at least in the year of study.

### 4.1 Mating system

Given the small size and other features of the two populations of *T. succulenta* on Graciosa island, the rigorous documentation of high outcrossing is rather surprising. The high self‐seed set of these plants in the greenhouse (the SC breeding system; Crawford et al., 2019; L. Borges‐Silva & M. Moura, unpubl.) suggests that these populations should be capable of selfing in the field. Selfing in the absence of pollinators in the greenhouse is likely facilitated by nyctinastic movements with capitula opening and closing daily during anthesis. Nyctinastic movements occur widely in Asteraceae and have been documented extensively in tribe Cichorieae, of which *Tolpis* is a member (Stirton, 1983). These movements have been noted in all species of *Tolpis* cultivated in the greenhouse, and they ostensibly bring the pollen in contact with the receptive style branches of florets in a capitulum (Crawford et al., 2019). Silva et al. (2016) suggested that the same mechanism may function in a rare species of *Sonchus* (tribe Cichorieae). Finally, Arista et al. (2017) hypothesized that nyctinastic movements function as a “delayed selfing mechanism” in *Hypochaeris salzmanniana* (Cichorieae). Delayed selfing, as opposed to prior selfing and competing selfing (see Lloyd, 1979), does not hinder outcrossing when pollinators are abundant. The high outcrossing in *T. succulenta* on Graciosa in 2015 may owe to the fact that pollinator activity was likely saturating during that year. Weissmann et al. (2017) reported that six wild bee species on Graciosa visit Asteraceae. In particular, pollinator observations on *T. succulenta* indicate visitation by many small halictid bees (field observations by H. Schaefer in August, 2018; discussion in Crawford et al., 2019), and flies are frequent visitors to *T. succulenta* in the Azores (L. Borges Silva, field observations). Interestingly, field observations, and biogeographic and genetic data suggest that halictid bees are recent introductions to the Azores (Weissmann et al., 2017). This would mean that the evolution of SC *Tolpis* on Graciosa took place in the absence of pollinating bees but that existing populations might now be strongly dependent on these presumably introduced pollinators. That said, *Tolpis* flowers, like other members of Azorean Asteraceae (e.g., *Solidago azorica*; Weissmann & Schaefer, 2017), are accessible to a broad range of generalist pollinator groups including dipterans, lepidopterans, and coleoptera, all of which have a number of native species in the Azores. Additional years of study within these populations are needed to determine whether delayed selfing provides reproductive assurance when pollinators are limited.

As indicated in the introduction, there are two prevalent nonmutually exclusive hypotheses for the evolution of selfing during the colonization and establishment of small founding populations such as *T. succulenta* on Graciosa. Reproductive assurance posits that selfing is selected when the paucity of compatible mates (e.g., Reinartz & Les, 1994; Young & Pickup, 2010) and/or pollinators (e.g., Busch & Delph, 2012; Goodwillie, 2001) reduce outcrossed seed production. It is possible that few functioning S‐alleles in the small populations of *T. succulenta* on Graciosa result in a paucity of compatible mates. Sporophytic self‐incompatibility (SSI), which is characteristic of Asteraceae (Crowe, 1954; Gerstel, 1950; Hughes & Babcock, 1950), may appear more restrictive in terms of self‐ and cross‐compatibility than gametophytic self‐incompatibility (GSI) systems. In the latter, the haploid genotype (allele at S‐locus) of pollen controls compatibility but with SSI the alleles in the parental plant occur on the pollen grain and if either of the pollen alleles are the same as the stigma, fertilization does not occur. However, dominance relationships among S‐alleles of SSI in Asteraceae (Crowe, 1954; Gerstel, 1950; Hughes & Babcock, 1950) increases cross‐ and self‐compatibility because recessive alleles are not expressed, and therefore do not prevent fertilization in the presence of more dominant alleles (Brennan et al., 2011, 2013; Hiscock, 2000). Alleles may also show different dominance relationships in the stigma and pollen (Brennan et al., 2006; Hiscock & Tab ah, 2003). Unlike codominant alleles where an increase in frequency of an allele will result in it finding fewer compatible mates in a population (Byers & Meagher, 1992), more recessive alleles in a dominance hierarchy are not subjected to negative frequency dependent selection and will increase in a population. Thus, species with SSI may set seed both by outcrossing and selfing in small populations despite low S‐allele diversity (Brennan et al., 2002; Silva et al., 2016).

The automatic selection hypothesis posits that SC mutations are selected because selfing variants, in contrast to outcrossers, can fertilize their own ovules giving them a 3:2 transmission advantage (Fisher, 1941; Pannell, 2015). The highly outcrossing mating system in the two populations of Azorean *Tolpis* despite having the ability to set self‐seed in the greenhouse indicates that there are negative factors associated with selfing, one of which could be inbreeding depression (ID), see below. Selfing may also be disadvantageous when there is a reduction in pollen and ovules available for outcrossing because they are used for selfing, so‐called pollen and seed discounting (Holsinger, 1991; Nagylaki, 1976). The mating system of plants where SC has ostensibly evolved recently may depend on a complex combination of the aforementioned factors (Voillemot et al., 2019). The role(s) of ID and pollen/seed discounting for the outcrossing mating system in the two SC populations of Azorean *Tolpis* are not known.

### 4.2 Inbreeding depression

ID is considered a major factor opposing the evolution of selfing subsequent to SI breakdown (Charlesworth & Willis, 2009). ID within populations can be estimated comparing the inbreeding coefficients of seeds and adults (Ritland, 1990; Scofield & Schultz, 2006) because it will reduce the mean inbreeding level of adults relative to seeds. The parents and progeny in both populations of *Tolpis succuletna* were highly outbred, and the results therefore not informative on the strength of ID. The highly outbred maternal plants indicate that outcrossing occurred in earlier generations, and the results for progeny grown from seed collected in 2015 are not an anomaly for that flowering season. Selfed seed from these populations are vigorous when grown under greenhouse conditions and these plants themselves produce high levels of self‐seed (D. J. Crawford et al., unpubl.). Clearly, more extensive experimental studies in the field and greenhouse are needed to estimate ID for *Tolpis*. Especially desirable would be studies of inbreeding depression in both cultivation and nature, as was done by Arista et al. (2017), who demonstrated higher ID in nature than in cultivation for *Hypochaeris salzmanniana* (like *Tolpis* a member of tribe Cichorieae). Arista et al. (2017) further demonstrated variation in self‐seed set and mating system in the same populations depending on pollinator availability. Low pollinator attendance and higher selfing were associated, with progeny from selfing exhibiting inbreeding depression in greenhouse and field studies. Arista et al. (2017) suggested that the mixed mating system in this species results from selection for reproductive assurance when pollinators are limiting and inbreeding depression maintaining outcrossing.

Studies of ostensibly recent transitions to selfing in natural populations and the experimental introduction of SC plants into SI populations show differing scenarios of how mating system responds to SC. Self‐incompatible populations of the perennial herb *Linaria cavanillesii* (Plantaginaceae) show high inbreeding depression, whereas inbreeding depression has been purged in SC populations (Voillemot & Pannell, 2017). However, the SC populations have a mixed mating system despite the absence of inbreeding depression and the presence of autonomous selfing (Voillemot & Pannell, 2017). Voillemot et al. (2019) introduced SC individuals of *L. cavanillesii* into SI populations of the same species and found a rapid spread of SC within the populations, driven by higher seed set and greater outcross siring. By contrast, the experimental results of Layman et al. (2017) for *Leavenworthia alabamica* (Brassicaceae) factors indicated that, despite the advantages of higher seed set in SC plants, seed discounting and inbreeding depression ostensibly prevent the establishment of SC mutations within populations and result in the persistence of SI populations. An experimental study of the same species by Baldwin and Schoen (2019) showed that inbreeding depression was largely unchanged following forced inbreeding of parental SI plants through three generations.

### 4.3 Paternity

Pannell and Labouche (2013) discussed the factors that influence polyandry, and particularly those determining the number of sires per maternal plant in small populations. A small number of compatible mates, discussed above, could reduce the number of potential sires (Hardy et al., 2004; Young & Pickup, 2010). Pollinator abundance and/or foraging behavior could impact sire number, with a paucity of pollinators and limited foraging distance reducing siring number. As in all Asteraceae, the inflorescence and floral morphology of *Tolpis* may affect the siring pattern. The close aggregation of small flowers in the capitulum could facilitate the pollination of many flowers by a single visit and result in correlated paternity. However, a single visit by a pollinator with pollen from prior visits to several plants (pollen carry over) could result in multiple paternity. The number of florets that can be pollinated per capitulum per visit depends on the number of open, receptive (in female phase) florets present in a capitulum at any given time. In some instances, individual florets may be receptive to pollen for only 1 or 2 days (Guerrina et al., 2016; Nielsen et al., 2000; Sun & Ganders, 1988). This temporal separation in receptivity should favor multiple paternity within a capitulum. The factors that could influence sire number are little known for insular *Tolpis*. However, individual capitula are open for at least 7 days but close nightly (Crawford et al., 2019; unpubl.), potentially facilitating pollination within a single capitulum over a relatively prolonged period. Multiple paternity could also be promoted by the nonoverlapping flowering periods of different capitula on the same plant. Once flowering commences on individuals of Azorean *T. succulenta*, there are several open capitula during much of the flowering period (D. J. Crawford et al., unpubl.). In the present study, seed was collected in bulk from each maternal plant, thus precluding determination of intra‐ and intercapitular components of multiple paternity.

The percent of full sibs detected in the present study may be compared with other Asteraceae, including the recent study of *T. succulenta* on Madeira island (Gibson et al., 2020). Those populations had 22% full sibs, some 50% higher than detected in the two small Graciosa populations. Thus, correlated paternity is higher in the SI species in Madeira than in the SC populations on Graciosa. The reason(s) for the differences are obscure and comments would be highly speculative. Hardy et al. (2004) found that in the rare endemic SI perennial herb *Centaurea corymbosa* (Asteraceae), 20% of sibs were full (same sire), and Sun and Ritland (1998) found 19% full sibs in the SI annual *Centaurea solstitials*. Perhaps, the important point is that in the small insular populations on Madeira and Graciosa, the number of sires among fruits of maternal plant is comparable not only to the other few Asteraceae investigated but also to among‐fruit values estimated for other plant families (Pannell & Labouche, 2013). There is little evidence that particular sires are contributing disproportionately to the progeny of maternal plants, making it unlikely that biparental inbreeding is occurring in the populations.

### 4.4 Summary and questions for future study

There are two major findings of this study. The first is that two small insular populations are highly outcrossing in nature despite the breakdown of SI. However, families of plants grown from seeds collected in nature yield high self‐seed set in the greenhouse. The origin of SC in these two small populations that have ostensibly recently colonized Graciosa would seem to favor the transition to selfing (Pannell, 2015). The reasons for the somewhat unexpected results remain to be determined, and in a real sense, this study raises more questions, than it answers. One likely reason for the retention of outcrossing is high ID (Layman et al., 2017; Pannell, 2015). That is, selfed seeds are presumably aborted on production, do not germinate well, do not flower well, or are vegetatively noncompetitive. Whether this is the situation for *T. succulenta* awaits further study; two generations of selfed progeny in the greenhouse flowered and set fruit but more thorough studies are needed. Of course, it may be that fitness of selfed progeny is lower in the natural habitat than under greenhouse cultivation (Arista et al., 2017; Armbruster & Reed, 2006). Self‐seed set is sometimes used to infer the breeding system in plants from oceanic archipelagos (Anderson et al., 2001; Bernardello et al., 2001; Chamorro et al., 2012; Crawford et al., 2011) and indeed likely provide useful first estimates of breeding system. However, the present study provides a caveat in making such inferences. Indeed, Crawford et al. (2019) appropriately concluded that the high self‐seed set in an experimental setting with no pollinators demonstrated SC, and a logical hypothesis emanating from those experiments is that the high self‐seed set in the two natural populations resulted from selfing. As an additional caveat, the first study of mating system in Macaronesian *Tolpis* using progeny arrays and allozyme markers showed that two of six populations of an ostensibly highly outcrossing (very low self‐seed set) species had mixed mating systems.

The second major result from this study is the multiple sires for all maternal plants in both populations. The markers and methods of analyses employed in the present study provide strong probabilistic assignment of progeny to different sires. The factors responsible for the low correlated paternity are not known, but possibilities include adequate pollinator visitation, pollen carry over, individual capitula receptive 7 days or more, and different capitula of each maternal plant open progressively over the flowering season.

Both results speak to the need for field studies over several seasons. How “typical” are the mating system estimates from 2015 over multiple seasons? If selfing is higher in some years, is this outcome correlated with changes in the extent of multiple paternity? Such a correlation might naturally emerge if higher selfing is caused by lower pollinator levels (and hence less frequent visitation). The present study indicates that both the maternal plants in the natural populations and their progeny are highly outbred, suggesting that our results apply across generations. Other observations within/over flowering seasons that could provide insights into the reproductive biology of the two populations include seed set; number of flowering plants and temporal variation in flowering; and abundance and types of floral visitors.

Gibson et al. (2020) discuss the advantages of the methods employed herein for studying the small populations of rare island plants. A major advantage, with conservation implications, is that mating system can be inferred because individual progeny can be called as selfed or outcrossed with few maternal families and few progeny per family. This advantage can hardly be overstated given the low seed set available for progeny arrays and small population sizes. Although small populations provide challenges for mating system and paternity studies, they may offer certain advantages using genome‐wide genotyping. In small populations such as the two examined in this study, it may be feasible to map all plants making it possible to detect genetic structure in the populations (Colicchio et al., 2020) and to infer not just the number of sires but the specific sires of progeny (Gibson et al., 2020).

## CONFLICT OF INTEREST

The authors declare no conflict of interest.

## AUTHOR CONTRIBUTIONS

**Daniel J. Crawford:** Conceptualization (equal); Investigation (equal); Project administration (equal); Writing‐original draft (equal); Writing‐review & editing (equal). **Benjamin Kerbs:** Conceptualization (equal); Data curation (equal); Formal analysis (lead); Visualization (lead); Writing‐review & editing (equal). **Mónica Moura:** Conceptualization (supporting); Investigation (supporting); Resources (equal); Writing‐review & editing (equal). **Lurdes Borges Silva:** Conceptualization (supporting); Investigation (supporting); Resources (equal); Writing‐review & editing (equal). **Hanno Schaefer:** Conceptualization (supporting); Investigation (supporting); Resources (equal); Writing‐review & editing (equal). **Griffin White:** Conceptualization (equal); Data curation (supporting); Writing‐review & editing (equal). **Keely Brown:** Conceptualization (supporting); Investigation (supporting); Methodology (equal); Writing‐review & editing (equal). **Mark E. Mort:** Funding acquisition (lead); Project administration (equal); Resources (equal). **John K. Kelly:** Conceptualization (lead); Data curation (lead); Formal analysis (equal); Funding acquisition (equal); Investigation (lead); Project administration (lead); Writing‐original draft (equal); Writing‐review & editing (equal).

## Supporting information

Supplementary Material

## Data Availability

The sequence data are deposited in the NCBI Sequence Read Archive (SAMN16395570–SAMN16395611) under BioProject ID PRJNA668054.
